# Biocontrol of Plant Diseases by Means of Antagonist Microorganisms, Biostimulants and Induced Resistance as Alternatives to Chemicals

**DOI:** 10.3390/plants11243521

**Published:** 2022-12-14

**Authors:** Eugenio Llorens, Carlos Agustí-Brisach

**Affiliations:** 1 Departament of Biology, Biochemistry and Natural Sciences, Universitat Jaume I de Castellón (UJI), 12006 Castellón de la Plana, Spain; 2 Department of Agronomy (DAUCO, Unit of Excellence María de Maeztu 2020-23), ETSIAM, University of Cordoba (UCO), 14071 Córdoba, Spain

Plant diseases are one of the biggest problems in conventional agriculture as they reduce both yield and crop value. Therefore, it is necessary to establish systems that protect against pests to maintain or improve yields while ensuring high food quality. The chemical pesticides used in conventional agriculture over the last 50 years contaminate the environment, including soil and water; may leave residues in food; and affect other beneficial organisms that are not targeted by treatment. Therefore, researchers are searching for natural and eco-friendly alternatives for crop protection that avoid the problems associated with chemical pesticides.

In recent decades, the natural compounds and beneficial microorganisms for use as biological control agents (BCAs) or plant biostimulators have attracted interest. These treatments have several advantages compared to classic pesticides; for example, they exhibit low toxicity, zero residue in foods, and their mode of action often allows their use to be preventive or curative. These advantages make them suitable for sustainable agriculture, for fulfilling new demands from the agro-food sector of the Euroregion, as well as for society in the frame of the European Green Deal.

The term biocontrol comprises a group of treatments that are based on natural compounds, extracts or microorganisms. These treatments have demonstrated effectiveness in protecting plants against both biotic and abiotic stresses. This protection can be achieved through several systems, depending on the BCA used, among which we highlight the use of antagonistic microorganisms and host resistance induction.

Antagonistic microorganisms can colonize the rhizosphere or the aerial parts of the plant without causing any damage to the plant, preventing colonization by other pathogenic microorganisms. These microorganisms, in addition to competing for the ecological niche, can release compounds into their environment with diverse functions. It has been demonstrated that some exudates are able to prevent the development of pathogens, providing partial protection to plants. On the other hand, some microorganisms also excrete molecules that improve plant growth and performance under stressful conditions, such as hormones and siderophores.

Plants also possess a response system that allows them to fight infection by pathogens. It has been shown that these responses can be enhanced through the application of certain beneficial compounds and microorganisms, resulting in a faster and stronger response. This enhanced response is often enough to reduce the infection below the economic threshold of damage. The activation of the plant’s immune system against subsequent stress can be also divided in two types. On the one hand, there is direct activation of the defensive mechanisms, in which the hormonal signaling pathways (mainly jasmonic acid and salicylic acid) activate the expression of genes and other defense mechanisms involved in the response. On the other hand, the enhancement of resistance can be a "priming" response. In this case, plants do not show a strong defensive response when the resistance inducer is applied, but primed plants show a faster and stronger defensive response upon exposure to stress.

This Special Issue highlights a selection of cutting-edge research on beneficial microorganisms, including cyanobacteria, actinomycetes, bacteria and fungi. Abdelaziz et al. [1] showed that foliar spraying applications of cyanobacteria were effective in relieving the toxic influences of *Fusarium oxysporum* on infected pepper plants. Elshafie and Camele [2], Carlucci et al. [3] and Diaz-Diaz et al. [4] demonstrated the effectiveness of actinobacterial strains (mainly *Streptomyces* spp.) in controlling soil-borne pathogens of tomato, fennel and bean crops, respectively. The effect of bacterial strains of *Bacillus velezensis* was evaluated, with successful results obtained against the nematode *Meloidogyne incognita* in cucumber plants by Asaturova et al. [5], and against several fungal diseases of grapevine by Hamaoka et al. [6]. López-Moral et al. [7] demonstrated the influence of several BCAs and biofertilizers on the effect of olive stem extracts on the viability of *Verticillium dahliae* conidia. The effectiveness of *Trichoderma* spp. strains as BCAs was also demonstrated against *Macrophomina phaseolina* in peanut crop by Martínez-Salgado et al. [8]. These articles reflect the potential of a broad diversity of microorganisms against a range of plant diseases.

Moreover, the selection of articles on biostimulation based on fertilizers, soil amendments or waste extracts provides a vision of how plants can be protected without the use of synthetic chemical pesticides. Likewise, Stavridou et al. [9] demonstrated that the use of biosolids for soil amendment had positive effects not only on plant health and protection, but also on the growth of non-pathogenic antagonistic microorganisms against *F. oxysporum* f. sp. *radicis-lycopersici* in the tomato rhizosphere. El Boumlasy et al. [10] showed the effectiveness of new natural substances, obtained from shrimp wastes using minimal processing, against fungi and oomycetes belonging to the genera *Alternaria*, *Colletotrichum*, *Fusarium*, *Penicillium*, *Plenodomus* and *Phytophthora*. A site-targeted copper-based biofertilizer was evaluated in combination with a commercial *Trichoderma atroviride* by Reis et al. [11], who reported that this combination may constitute a promising long-term approach to mitigating the impact of Botryosphaeria dieback in grapevine.

Finally, Liu et al. [12] reviewed the literature on the role of Cruciferae glucosinolates in resistance against diseases and pests. This review explores the mechanisms via which glucosinolates act as a defensive substance, participate in responses to biotic stress, and enhance plant tolerance to stress.

We are grateful to the co-authors who have contributed to this Special Issue and hope that the knowledge provided in the published papers will encourage the use of eco-friendly management strategies for protection against plant diseases.

## Data Availability

Not applicable.
